# Anti-inflammatory and antioxidative effects of genistein in a model of spinal cord injury in rats

**DOI:** 10.2478/abm-2021-0029

**Published:** 2021-10-29

**Authors:** Ercan Bal, Şahin Hanalioğlu, Aydın Sinan Apaydın, Ceylan Bal, Almila Şenat, Berrak Gümüşkaya Öcal, Burak Bahadır, Ömer Faruk Türkoğlu

**Affiliations:** Department of Neurosurgery, Ankara Yıldırım Beyazıt University, School of Medicine, Ankara 06760, Turkey; Department of Neurosurgery, Ankara City Hospital, Ankara 06800, Turkey; Department of Neurosurgery, Ankara Hacettepei, University, School of Medicine, Ankara 06230, Turkey; Department of Neurosurgery, Ankara Yıldırım Beyazıt University, School of Medicine, Ankara 06760, Turkey; Department of Pathology, Ankara Yıldırım Beyazıt University, School of Medicine, Ankara 06760, Turkey

**Keywords:** antioxidants, genistein, oxidative stress, spinal cord injuries, sulfhydryl compounds

## Abstract

**Background:**

Neurological damage from spinal cord injury (SCI) is a result of primary mechanical injury and secondary damage from oxidative stress and neuroinflammation. Although genistein has been shown to have potent antioxidant and anti-inflammatory effects in studies of brain injury, its effect on secondary damage in SCI has remained unknown.

**Objective:**

To determine effects of genistein in a model of SCI in rats.

**Methods:**

We divided 21 rats evenly into 3 groups, a control group, in which only a laminectomy was performed; a trauma group in which SCI was induced; and a genistein group in which genistein was administered subcutaneously after SCI. The rats were assessed using a Basso–Beattie and Bresnahan functional score at the 12th hour and on the 1st, 3rd, 5th, and 7th days. Biochemical analyses were conducted at the same time points to determine the serum levels of catalase, ischemia-modified albumin (IMA), disulfide (SS), total thiol (TT), native thiol (NT), disulfide/total thiol (SS/TT), and native thiol/total thiol (NT/TT). Total oxidant and antioxidant capacity, and oxidative stress index were determined in spinal cord tissue obtained on the 7th day together with immunohistochemistry for cyclooxygenase-2 levels.

**Result:**

Catalase activity on the 7th day was significantly (*P* = 0.001) higher in the genistein-treated rats than in other groups, and IMA levels became stable earlier (3rd day) in the genistein group. SS values were significantly (*P* = 0.004) lower in the genistein group. NT/TT ratio were significantly (*P* = 0.049) higher in the genistein-treated rats on the 7th day.

**Conclusion:**

Genistein has antioxidant, anti-inflammatory, and protective effects in a model of SCI in rats and warrants further study.

Neurological damage from spinal cord injury (SCI) is a result of primary mechanical damage and subsequent secondary damage [1,2,3,4]. Oxidative stress occurs during primary damage due to the cellular release of cytoplasmic components and mitochondrial dysfunction and continues throughout secondary damage due to neuroinflammation. Secondary damage includes tissue necrosis from free oxygen radicals, lipid peroxidation products, immune responses, and a complex series of events involving energy metabolism. Oxygen radicals such as O^−^ and OH− ions are highly active free radicals that are usually eliminated after being converted into O_2_ and H_2_O_2_ via antioxidant enzymes such as superoxide dismutase, glutathione peroxidase, and catalase. Oxidative stress creates an imbalance between oxidants and antioxidants at the cellular level. An antioxidant defense system protects cells by neutralizing the harmful effects of oxidants and free radicals and high levels of antioxidants have been found in the spinal cord [5, 6]. If an appropriate antioxidant response is not obtained following SCI, further cellular damage occurs, contributing to a positive feedback cycle resulting in further oxidative stress [5]. Oxidative stress is an ongoing process throughout both primary and secondary damage, and the severity and uncontrolled nature of oxidative stress are related to the magnitude of the damage [1, 4,5,6]. Thus, markers of oxidative stress have been used to monitor SCI and response to treatment [7,8,9], and many studies have focused on resolving inflammation and antioxidant therapies for traumatic brain and SCI [6, 10].

Genistein, a phytoestrogen, is an isoflavonoid found in high concentrations in soybeans [11,12,13]. Genistein has been shown to reduce the risk of cancers such as those of the breast and prostate, which are associated with estrogen [11]. In addition, positive effects on bone mineral development, anti-menopausal effects, and antioxidant and anti-inflammatory effects have been reported [13,14,15,16,17,18,19,20]. Although genistein has been shown to have potent antioxidant and anti-inflammatory effects in several studies of brain injury [16, 17], to our knowledge, its effect on SCI-induced damage remains unknown. In the present study, the antioxidant and anti-inflammatory effects of genistein were investigated in a model of SCI in rats, and its contribution to the recovery of the spinal cord was evaluated biochemically, histopathologically, and functionally through oxidant and antioxidant markers.

## Methods

The protocols for this study were approved by the local ethics committee of Kobay Deney Experimental Animals Laboratory (No. 280). All protocols followed national guidelines for the care and use of laboratory animals and were compliant with the U.S. Health Research Extension Act (Public Law 99–158, 1985 “Animals in Research”). Reporting follows ARRIVE 2.0 guidelines [21]. Adult female Wistar–Hannover albino rats (each weighing 250–300 g; n = 21) were separated into 3 groups of 7 rats without selection. During the experiments, all rats were kept in a standard postoperative care room (at 20–25 °C with 50%–60% humidity grouped in polycarbonate cages) and allowed standard feed, under 12-h light and dark cycles.

A commercial kit was used to determine the total anti-oxidant status (TAS) and total oxidative status (TOS) in the tissue [22, 23]. Tissue protein was assayed using the Lowry method [24]. Oxidative stress index (OSI) was obtained by dividing TOS values by TAS values [OSI (arbitrary Unit) = (TOS, μmol H_2_O_2_ eq/L)/(TAS, μmol Trolox eq/L)].

Serum thiol–disulfide (SS) was measured with an automatic analyzer (Cobas 501; Roche) using a method developed by Erel and Neselioglu [25]. Briefly, disulfide bonds in the sample were converted into functional thiol groups with NaBH_4_. The total thiol (TT) content in the sample was calculated using a modified Ellman reagent. Serum disulfide amount was determined using the following formula: (serum TT − serum native thiol [NT])/2.

Serum ischemia-modified albumin (IMA) levels were determined according to the Bar–Or method [26]. The results are expressed in absorbance units (AbsU). Serum catalase activity was measured using a method described by Góth [27].

### Experiment

**Figure 1:**
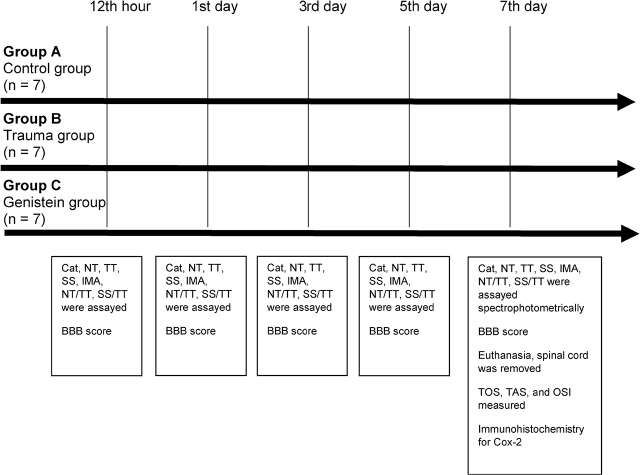
Timeline for the experiment. In group A (n = 7, control group, only T7-8-9 laminectomy), in group B (n = 7, trauma group, after T7-8-9 laminectomy and trauma was induced with a modified Allen weight-drop method, in group C (n = 7, genistein group, T7-8-9 laminectomy and spinal trauma was induced as described above. It was planned that group C would receive the first dose of genistein at 15 min immediately after trauma and would be given genistein subcutaneously at 2 mg/kg/day for 7 days). The rats were killed on the 7th day and the spinal cord was removed as a whole. No rats were lost, and no infection, or additional problems were observed during the experiment. cat, catalase; Cox-2, cyclooxygenase-2; IMA, ischemia-modified albumin; NT, native thiol; OSI, oxidative stress index; SS, disulfide; TT, total thiol; TAS, total antioxidant status; TOS, total oxidative status.

The rats were separated into 3 equal number groups without selection (A: control group, B: weight-drop group, C: genistein group) (**Figure 1**). After 6 h of fasting, 1 mg/kg of ketamine (Ketalar, Pfizer) and 1 mg/kg of xylazine (Alfazyne, Alfasan) were administered intraperitoneally. The rats were allowed spontaneous breathing, and T7-8-9 laminectomies were conducted in accordance with standard surgical procedures. Analgesic agents were not used during the recovery process because we sought to evaluate anti-inflammatory markers.

- In the control group (n = 7), only a T7-8-9 laminectomy was performed.
- In the trauma group (n = 7), after a T7-8-9 laminectomy, trauma was induced with a modified Allen weight-drop apparatus. Using a glass tube with a diameter of 0.5 cm and a length of 10 cm, a 10 g weight was dropped on the intact dura from a height of 10 cm.
- In the genistein group (n = 7), a T7-8-9 laminectomy was performed, and spinal trauma was induced as described above. Rats in this group were administered a first dose of genistein (Sigma-Aldrich; 2 mg/kg subcutaneously) at 15 min immediately after trauma and further administration of genistein at 2 mg/kg/day for 7 days.

After deep general anesthesia with 1 mg/kg of ketamine and 1 mg/kg of xylazine the rats were killed on the 7th day by transcardiac perfusion with saline followed by phosphate-buffered 4% paraformaldehyde (in 0.1 mol/L phosphate-buffered saline, pH 7.4), and their spinal cords removed. No rats died unintentionally, and no infection or serious adverse events were observed during the experiment.

#### Biochemical analysis

Tail blood was taken before anesthesia, at the 12th hour, and on the 1st, 3rd, 5th, and 7th days after the surgical procedure. Catalase, IMA, and thiol–disulfide balance were assayed. After killing the rats on the 7th day, the spinal cord tissue in the T7-8-9 region was removed, and TOS and antioxidant capacity (TAS), and OSI determined in the spinal cord tissue samples.

#### Functional analysis

Basso–Beattie and Bresnahan (BBB) scores were examined in all rats at baseline and the 12th hour, and on the 1st, 3rd, 5th, and 7th days. The results were evaluated statistically [28, 29].

#### Histopathology

After the rats were killed on the 7th day, their entire spinal cords were removed. Next, 1-cm specimens from the traumatized site of each spinal cord were obtained and put into phosphate-buffered 10% formaldehyde. On gross dissection, sections of spinal cords cut perpendicularly were processed and embedded in paraffin blocks. Then, 4-μm sections were deparaffinized in xylene and rehydrated through a graded series of ethanol before hematoxylin and eosin staining and immunohistochemistry.

Immunohistochemistry was performed using a mouse monoclonal cyclo-oxygenase-2 (Cox-2) antibody (clone D-12: catalog No. sc-166475, Santa Cruz Biotechnology; Research Resource Identifier (RRID): AB_2276666) primary antibody with a Leica Bond-Max auto-stain detection system (catalog No. DS9800, Leica) according to the manufacturer’s instructions.

A pathologist, who was blinded to the groups, examined the immunohistochemically stained sections. Cox-2 immunoreactive (IR) cells were counted per 3 high powered fields (HPFs Olympus BX51 microscope 400×) for each spinal cord.

### Statistical analyses

Data were analyzed using IBM SPSS Statistics for Windows (version 25). Data are expressed as mean ± standard deviation (SD) or median (interquartile range; Q1, Q3). A one-way analysis of variance (ANOVA) followed by a Bonferroni post hoc test and a Kruskal–Wallis test followed by a Dunn post hoc test were used to analyze differences between independent variables. A repeated measures ANOVA and Friedman analyses with Bonferroni post hoc correction were used to determine the differences between the dependent variables. Differences with *P* < 0.05 were considered as significant.

## Results

### Biochemical findings

#### Catalase

**Figure 2:**
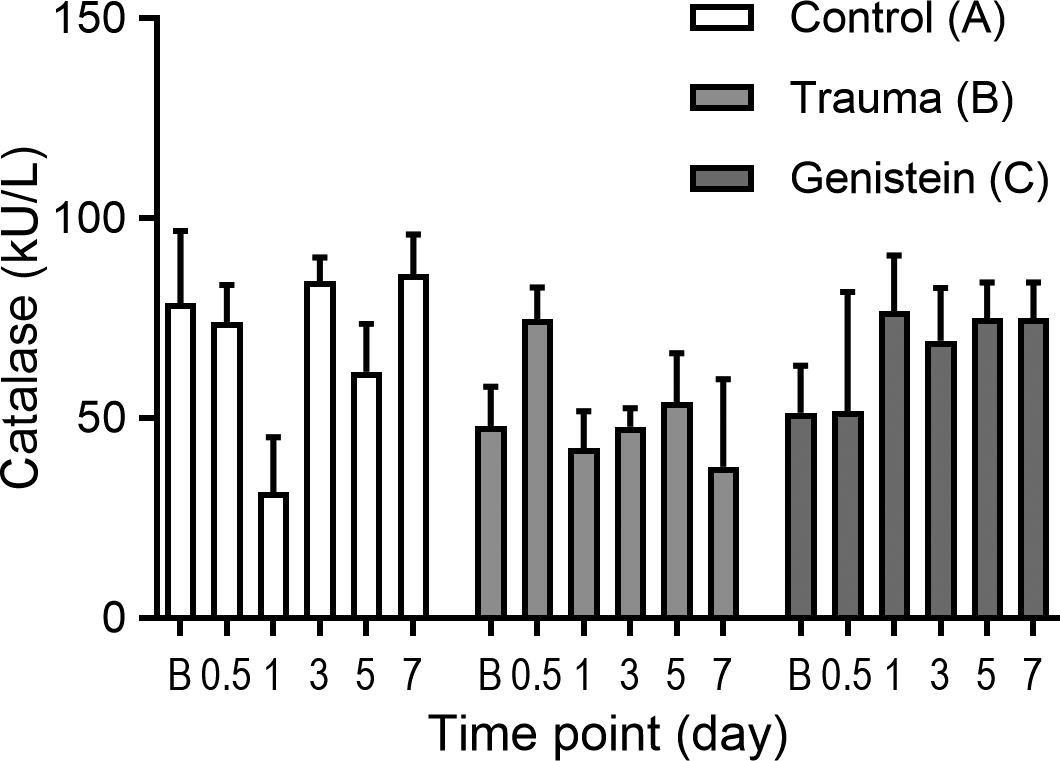
Catalase serum levels. Significant difference was observed on the 7th day (*P* = 0.001). Compared with the baseline, serum catalase values increased slightly in the control group (A white bars), decreased in the trauma group (B light gray bars), and increased significantly in the genistein group (C dark gray bars) on the 7th day. B, baseline; 0.5, 12th hour. Bars indicate means. Error bars (SD).

There was no significant difference in the baseline serum activity between the groups. Although no significant difference (*P* = 0.109) was found between the groups at the 12th hour, on the 1st day the activity in the control group (A) was significantly lower (*P* = 0.001) than that in the trauma group (B) and genistein group (C), and activity in group B was significantly lower (*P* = 0.001) than that in group C. Compared with the baseline, catalase activity was decreased slightly in group A, increased slightly in group C, and increased in group B at the 12th hour. On the 1st day, catalase activity decreased in groups A and B and increased in group C, and was significantly different between the groups (*P* = 0.001). A significant difference (*P* = 0.001) was also found on the 7th day. Compared with the baseline, catalase activity increased slightly in group A, decreased in group B, and increased significantly in group C on the 7th day (**Table 1, Figure 2**).

**Table 1:** Time-dependent comparison of oxidative and antioxidative serum markers between groups

**Parameter**	**Group**	**Time**						***P***

		**Baseline**	**12th hour**	**1st day**	**3rd day**	**5th day**	**7th day**	
Catalase (kU/L)								
	A	79.0 ± 18.0	74.2 ± 9.2	31.5 ± 13.8	84.3 ± 6.1	61.7 ± 12.0	86.0 ± 10.0	<0.001
	B	48.1 ± 9.9	74.9 ± 7.9	42.6 ± 9.2	48.0 ± 4.6	54.1 ± 12.3	37.9 ± 21.9	0.02
	C	51.4 ± 11.9	51.8 ± 30.0	76.9 ± 13.9	69.4 ± 13.3	75.0 ± 9.1	75.0 ± 9.1	0.002
	*P*	0.11	0.11	0.001 (x, z)	0.15 (x, y, z)	0.15	0.001 (x, z)	
Ischemia-modified albumin (AbsU)								
	A	0.84 ± 0.05	0.82 ± 0.05	0.99 ± 0.05	0.93 ± 0.04	0.91 ± 0.05	1.01 ± 0.03	<0.001
	B	0.87 ± 0.04	0.93 ± 0.03	0.91 ± 0.03	0.78 ± 0.08	0.92 ± 0.04	0.92 ± 0.03	0.001
	C	0.91 ± 0.03	0.92 ± 0.04	0.85 ± 0.04	1.00 ± 0.04	0.98 ± 0.05	1.07 ± 0.09	<0.001
	*P*	0.10	0.001 (x, y)	<0.001 (x, y, z)	<0.001 (x, y, z)	0.10	0.004 (x, z)	
NT (mmol/L)								
	A	183.1 ± 37.8	110.9 ± 36.0	116.0 ± 16.2	71.7 ± 13.3	157.3 ± 26.0	87.4 ± 20.4	<0.001
	B	190.8 ± 51.0	222.2 ± 27.1	96.0 ± 31.4	127.8 ± 24.3	130.7 ± 29.8	166.3 ± 22.5	<0.001
	C	194.7 ± 38.7	99.9 ± 37.6	167.3 ± 24.7	99.8 ± 32.4	108.1 ± 28.6	71.2 ± 22.9	<0.001
	*P*	0.91	0.007 (x, y)	0.002 (y, z)	0.006 (x)	0.02 (y)	0.001 (x, z)	
TT (mmol/L)								
	A	250.3 ± 53.0	189.4 ± 47.5	158.5 ± 18.2	142.4 ± 12.5	202.5 ± 33.0	152.3 ± 21.0	0.002
	B	248.1 ± 63.7	281.7 ± 29.4	182.8 ± 54.5	207.4 ± 31.4	213.0 ± 42.6	238.4 ± 40.4	0.02
	C	245.0 ± 55.7	170.8 ± 23.0	210.4 ± 28.2	160.1 ± 40.3	187.7 ± 42.9	106.0 ± 19.7	0.02
	*P*	0.99	0.007 (x, z)	0.04 (y)	0.01 (x)	0.51	<0.001 (x, y, z)	
SS (mmol/L)								
	A	33.6 ± 10.2	39.2 ± 9.5	21.2 ± 6.1	35.3 ± 5.2	22.6 ± 5.0	32.4 ± 8.4	0.007
	B	28.6 ± 11.3	29.8 ± 8.8	43.4 ± 14.0	39.8 ± 12.5	41.2 ± 17.6	36.1 ± 11.7	0.33
	C	25.2 ± 10.5	35.5 ± 18.4	21.6 ± 4.2	30.2 ± 8.7	39.8 ± 9.6	17.4 ± 8.5	0.005
	*P*	0.297	0.286	0.004 (x, z)	0.193	0.014 (x, y)	0.004 (x, z)	
NT/TT (%)								
	A	73.4 ± 5.0	57.9 ± 6.7	73.3 ± 6.9	50.2 ± 7.3	77.7 ± 3.0	57.4 ± 9.8	<0.001
	B	77.1 ± 5.6	78.9 ± 5.7	52.5 ± 7.5	62.0 ± 9.8	62.1 ± 12.3	70.2 ± 5.7	<0.001
	C	70.0 ± 4.9	58.6 ± 18.9	79.5 ± 3.1	61.6 ± 11.5	57.4 ± 6.5	66.9 ± 18.5	<0.001
	*P*	0.12	0.003 (x, z)	0.001 (x, z)	0.04 (x, y)	0.005 (x, y)	0.049 (x)	
SS/TT (%)								
	A	13.3 ± 2.5	21.0 ± 3.4	13.3 ± 3.5	24.9 ± 3.7	11.2 ± 1.5	21.3 ± 4.9	<0.001
	B	11.5 ± 2.8	10.5 ± 2.8	23.8 ± 3.7	19.0 ± 4.9	18.9 ± 6.2	14.9 ± 2.9	<0.001
	C	10.0 ± 2.5	20.7 ± 9.5	10.3 ± 1.5	19.2 ± 5.7	21.3 ± 3.2	16.6 ± 9.2	<0.001
	*P*	0.11	0.003 (x, z)	0.001 (x, z)	0.04 (x, y)	0.005 (x, y)	0.049 (x)	
SS/NT (%)								
	A	18.4 ± 4.7	37.3 ± 9.8	18.8 ± 7.0	51.7 ± 16.3	14.5 ± 2.5	39.7 ± 17.6	<0.001
	B	15.2 ± 5.1	13.6 ± 4.6	46.9 ± 13.5	32.6 ± 13.9	33.5 ± 18.5	21.6 ± 5.6	<0.001
	C	12.7 ± 3.7	45.2 ± 36.4	13.0 ± 2.5	34.6 ± 22.1	38.2 ± 10.4	31.1 ± 28.5	0.001
	*P*	0.12	0.003 (x, z)	0.001 (x, z)	0.04 (x, y)	0.005 (x, y)	0.052 (x)	

Group A, control group; group B, trauma group; group C, genistein group; x, significant difference between groups A and B; y, significant difference between groups A and C; z, significant difference between groups B and C; *P*, a one-way ANOVA followed by a Bonferroni post hoc test and a Kruskal–Wallis test followed by a Dunn post hoc test were used to determine differences between independent variables. A repeated measures ANOVA and Friedman analyses with Bonferroni post hoc correction were used to determine differences between dependent variables. *P* < 0.05 was considered significant.

AbsU, absorbance units; NT, native thiol; NT/TT, native thiol/total thiol; SS, disulfide; SS/TT, disulfide/total thiol; TT, total thiol.

#### IMA

No significant difference in serum levels of IMA was observed between the 3 groups at baseline (*P* > 0.05). At the 12th hour, the level in the control group (A) was significantly lower (*P* = 0.001) than those in groups B and C, whereas there was no significant difference in levels between groups B and C. At the 12th hour, the IMA level had decreased in group A, whereas it had increased slightly in groups B and C. The lower level in group A compared with levels in groups B and C at the 12th hour, increased to be significantly higher (*P* < 0.001) than those in groups B and C on the 1st day. A comparison of the IMA levels at the 12th hour and on the 7th day after trauma demonstrated that there was no significant difference in the levels in groups A and B; however, the level in group C was significantly higher (*P* = 0.004) on the 7th day. In group B, IMA plateaued on the 5th day, whereas it plateaued on the 3rd day in groups A and C (**Table 1**).

#### Disulfide (SS)

**Figure 3:**
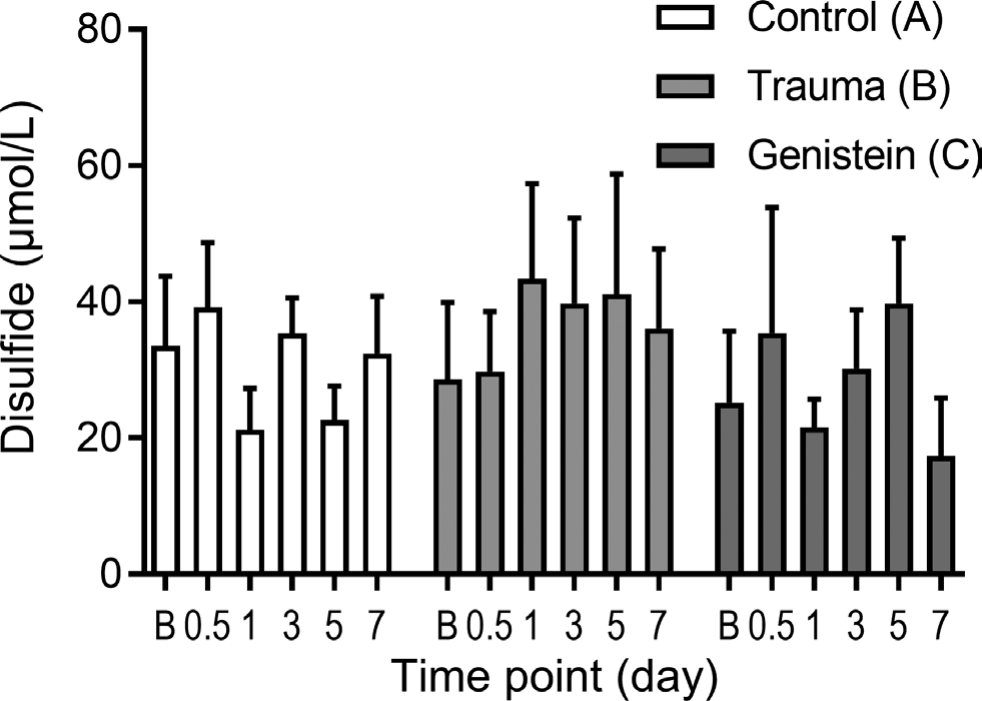
SS serum levels. Significant difference was observed on the 1st, 5th, and 7th day between the control group (A white bars) and trauma group (B light gray bars) and genistein group (C dark gray bars), and between groups B and C (*P* = 0.004, *P* = 0.014, *P* = 0.004, respectively). Compared with the levels at baseline, on the 7th day there was no significant difference in group A; an increase in group B; and a significant decrease in group C. Bars indicate means. Error bars (SD). SS, disulfide

There was no significant difference in the baseline serum SS serum levels between the groups. No significant difference (*P* = 0.29) was found between the levels at the 12th hour. A significant difference was observed on the 1st, 5th, and 7th days between the control group (A) (lower) and trauma group (B) (*P* = 0.004) and genistein group (C) (*P* = 0.014), and between groups B (higher) and C (*P* = 0.004). Compared with the baseline, on the 7th day there was no significant difference in the SS levels in group A, whereas we observed a significant increase in group B, and a significant decrease in group C (**Table 1, Figure 3**).

#### Native thiol (NT)

There was no significant difference in the baseline serum levels of NT between the groups. The level of NT in group C was significantly lower than those of groups A and B at the 12th hour (*P* = 0.007) and higher on the 1st day (*P* = 0.002) and lower on the 7th day (*P* = 0.001). NT levels fluctuated with temporal increases and decreases (**Table 1**).

#### Total thiol (TT)

There was no significant difference in the baseline TT serum levels between the groups. A significant difference in TT values was observed between the groups at the 12th hour (*P* = 0.007), 1st day (*P* = 0.04), and 7th day (*P* < 0.001). On the 7th day, the level of TT was significantly lower in the genistein group (C) than it was in the control (A) and trauma (B) groups, and lower in group A than in group B, which had the highest level of TT (*P* < 0.001). TT levels fluctuated with temporal increases and decreases (**Table 1**).

#### Native thiol/total thiol (NT/TT)

No significant difference in ratios was observed between the groups at baseline. There was a significant difference between the groups at all time points (12th hour, *P* = 0.003; 1st day, *P* = 0.001; 3rd day, *P* = 0.04; 5th day, *P* = 0.005; 7th day, *P* = 0.049). There was no significant difference in the control group (A) on the 7th day compared with the ratio at the 12th hour, whereas there was a decrease in the trauma group (B) and an increase in the genistein group (C) (**Table 1**).

#### Disulfide/total thiol (SS/TT)

No significant difference in the ratio was observed between the groups at baseline. There was a significant difference in the ratio between the groups at all time points (12th hour, *P* = 0.003; 1st day, *P* = 0.001; 3rd day, *P* = 0.04; 5th day, *P* = 0.005; 7th day, *P* = 0.049). Compared with the baseline, although an increase in the ratio was found on the 7th day for all groups, there was no significant difference from the ratio at the 12th hour in the control group (A); whereas, an increase in the ratio was found in the trauma group (B), and a decrease in the genistein group (C) (**Table 1**).

#### TAS, TOS, and OSI

These parameters were evaluated in the spinal cord tissue obtained on the 7th day. There was no significant difference between the groups for TAS (*P* = 0.26), TOS (*P* = 0.40), or OSI (*P* = 0.56) (**Table 2**).

**Table 2:** Time-dependent comparison of TAS, TOS, OSI, and Cox-2 immunoreactivity between the groups

	**Control group (A)**	**Trauma group (B)**	**Genistein group (C)**	***P***
TAS (nmol Trolox Eq/mg protein)	35.8 (30.7–40.8)	32.7 (28.6–36.8)	30.8 (29.8–35.3)	0.26
TOS (nmol H_2_O_2_ Eq/mg protein)	1.08 (0.59–1.12)	0.67 (0.58–0.85)	0.83 (0.60–1.11)	0.40
OSI (TOS/TAS)	0.03 ± 0.01	0.02 ± 0.01	0.03 ± 0.01	0.56
Cox 2 IR cells/3 HPFs	19.1 ± 5.3	17.1 ± 5.9	7.71 ± 5.0	0.008 (y, z)

Data are expressed as mean ± SD or median (Q1, Q3). Cox-2, cyclooxygenase-2; HPF, 400× high-powered field; IR, immunoreactive; OSI, oxidative stress index; TAS, total antioxidant status; TOS, total oxidative status; x, significant difference between groups A and B; y, significant difference between groups A and C; z, significant difference between groups B and C. Repeated measures ANOVA and Friedman analyses for (dependent) variables within the groups are indi cated as follows (**Table 1**).

#### Catalase

Group A: serum catalase activity on the 1st day was significantly lower than that at baseline, at the 12th hour, and activity on the 3rd and 7th days. Activity on the 5th day was significantly lower than that on the 3rd and 7th days. Group B: the activity at the 12th hour was significantly higher than that at baseline, and on the 1st, 3rd, 5th, and 7th days. Group C: the activity at baseline and at the 12th hour was significantly lower than that on the 1st, 3rd, 5th, and 7th days (**Figure 2**).

#### IMA

Group A: baseline and 12th hour levels were significantly lower than those on the 1st, 3rd, 5th, and 7th days. Group B: the IMA level on the 3rd day was significantly lower than that on the 12th hour, 1st, 5th, and 7th days. Group C: the level on the 1st day was significantly lower than that on the 3rd, 5th, and 7th days.

#### Native thiol

Group A: the serum NT level at baseline was significantly higher than that at the 12th hour, and on the 1st, 3rd, and 7th days. The level on the 3rd day was significantly lower than that at the 12th hour, and on the 1st and 5th days. The level on the 5th day was significantly higher than that on the 7th day. Group B: the levels at baseline and at the 12th hour were significantly higher than those on the 1st, 3rd, and 5th days. The level on the 1st day was significantly higher than those on the 3rd, 5th, and 7th days. Group C: the serum NT level on the 7th day was significantly lower than that at baseline and on the 1st day.

#### Total thiol

Group A: the serum level of TT at baseline was significantly higher than that at the 12th hour, and on the 1st, 3rd, 5th, and 7th days. The level on the 3rd day was significantly lower than that on the 5th day. Group B: the level at the 12th hour was significantly higher than that on the 1st and 3rd days. Group C: the serum level of TT on the 7th day was significantly lower than that at baseline, at the 12th hour, and the levels on the 1st, 3rd, and 5th days.

#### Disulfide

Group A: the level of serum disulfide at the 12th hour was significantly higher than that on the 1st and 5th days. Group B: no significant temporal differences in serum disulfide levels were found. Group C: the level on the 5th day was significantly higher than that on the 1st and 7th days (**Figure 3**).

#### NT/TT

Group A: the ratio at the 12th hour, and those on the 3th and 7th days were significantly lower than those at baseline, and on the 1st and 7th days. Group B: the ratio on the 1st day was significantly lower than that at baseline and at the 12th hour. Group C: the ratios at the 12th hour and on the 5th day were significantly lower than those at baseline and on the 1st day.

#### SS/TT

Group A: the ratio on the 3rd day was significantly higher than those at baseline, and on the 1st and 5th days. The ratio on the 5th day was significantly lower than that at the 12th hour and on the 7th day. Group B: the ratio on the 1st day was significantly higher than that at baseline, at the 12th hour, and on the 7th day. Group C: the ratio at the 12th hour was significantly higher than that at baseline and on the 1st day.

#### SS/NT

Group A: the ratio on the 3rd day was significantly higher than that at baseline, and on the 1st and 5th days. The ratio on the 5th day was significantly lower than that at the 12th hour and on the 7th day. Group B: the ratio on the 1st day was significantly higher than that at baseline, at the 12th hour, and on the 7th day. Group C: the ratio at the 12th hour was significantly higher than that at baseline and on the 1st day.

### Immunohistopathology

**Figure 4:**
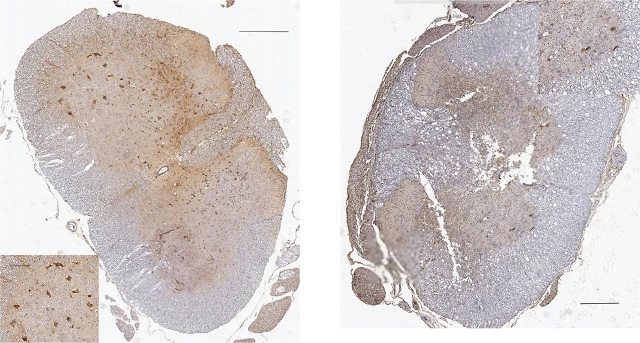
Immunohistochemically stained rat spinal cord sections with hematoxylin and eosin counterstain. A mouse monoclonal Cox-2 antibody (clone D-12: catalog No. sc-166475, Santa Cruz Biotechnology; RRID: AB_2276666) was used for primary detection, the monoclonal antibody was visualized using a diaminobenzidine chromogen system showing brown staining. Cox-2 positive cells were counted per 3 HPFs for each spinal cord (left panel, trauma group (B), right panel, genistein group (C). Left panel scale bar indicates 0.5 mm, inset scale bar indicates 50 μm. Right panel scale bar indicates 0.5 mm. Cox-2, cyclo-oxygenase-2; HPFs, 400× high-powered fields; RRID, research resource identifier.

**Figure 5:**
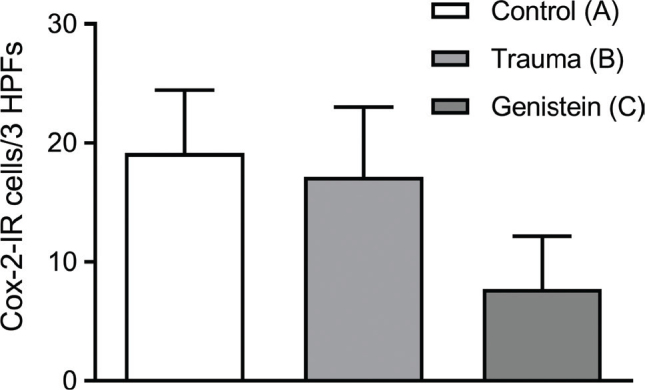
Cox-2 immunoreactivity scoring. Significant difference (*P* = 0.008) was found between the genistein (C) and other groups (A, B). The highest number of Cox-2 IR cells per 3 HPFs was observed in the control group (A white bar) and the trauma group (B light gray bar). The lowest number of Cox-2-IR cells was observed in the genistein group (C dark gray bar). Bars indicate means. Error bars (SD). Cox-2, cyclo-oxygenase-2; HPFs, 400× high-powered fields; IR, immunoreactive.

A significant difference was found between the groups (*P* = 0.008). The highest numbers of Cox-2 IR cells per 3 HPFs were observed in the control (A) and trauma (B) groups. The lowest numbers of Cox-2 IR cells per 3 HPFs were observed in the genistein group (C) (**Table 2, Figures 4** and **5**).

### Functional findings

At the 12th hour, the trauma (B) and genistein (C) groups had significantly (*P* = 0.006) poorer BBB scores than the control group (A). On the 1st day, the scores in the control group (A) were significantly higher (*P* = 0.002) than those in groups B and C, whereas there was no significant difference between groups B and C (*P* > 0.05). The scores in the genistein group (C) were significantly higher than those in the trauma group (B) on the 3rd (*P* = 0.001), 5th (*P* < 0.001), and 7th days (*P* < 0.001). The significantly higher score in the control group (A) compared with the score in the genistein group (C) found at the 12th hour and 1st day, was not found on the 5th or 7th days (*P* > 0.05) (**Table 3**).

**Table 3:** Time-dependent comparison of BBB scores in rats

**Time point**	**Control group (A)**	**Trauma group (B)**	**Genistien group (C)**	***P***
12th hour	7 (6–7)	5 (3–6)	5 (2–6)	0.006 (x, y)
1st day	13 (12–13)	11 (9–12)	11 (10–12)	0.002 (x, y)
3rd day	13 (13–13)	11 (9–12)	12 (11–13)	0.001 (x, y, z)
5th day	13 (13–13)	11 (9–12)	13 (12–13)	<0.001 (x, z)
7th day	21 (21–21)	18 (14–19)	21 (21–21)	<0.001 (x, z)
*P*	<0.001	<0.001	<0.001	

Data are expressed median (interquartile range; Q1, Q3). x, significant difference between groups A and B; y, significant difference between groups A and C; z, significant difference between groups B and C).

BBB, Basso–Beattie and Bresnahan.

Friedman analyses of BBB scores were as follows. Group A: 12th hour score was significantly lower than that on the 1st, 3rd, 5th, and 7th days. The score on the 7th day was significantly higher than that on the 1st, 3rd, and 5th days. Group B: 12th hour score was significantly lower than that on the 1st, 3rd, 5th, and 7th days. The score on the 7th day was significantly higher than that on the 1st, 3rd, 5th days. Group C: 12th hour score was lower than that on the 1st, 3rd, 5th, and 7th days. The score on the 7th day was significantly higher than that on the 1st, 3rd, 5th days (**Table 3**).

## Discussion

In the present study, although there was no difference between the groups at the 12th hour (*P* > 0.05), significant differences were noted in favor of the genistein group after the 1st day (*P* = 0.001). Compared with baseline, the highest increase in serum catalase level was observed in the genistein group on the 7th day. Treatment with genistein was not associated with significant change during the early period (12th hour), but was associated with a significantly higher catalase level on the 7th day (**Table 1, Figure 2**). Considering catalase levels, antioxidant capacity increased in the genistein group. A low level of catalase has been reported in spinal cord injuries, and catalase has been used to monitor treatment response [30,31,32].

IMA has been considered as a marker of oxidative processes. Oxygen radicals resulting from trauma damage the *N*-terminal of albumin under ischemic conditions and form a variant protein known as IMA. Clinical studies have found that its levels increase 6–12 h after an ischemic event, and return to baseline after 24 h [33, 34]. IMA can be used with high sensitivity and specificity in predicting mortality and damage in severe traumatic brain injury [34]. Because free oxygen radicals occur as a result of oxidative stress in secondary damage after SCI, it is believed that IMA levels may also be used as an early indicator of the damage [35]. In the present study, when the trauma and control groups were compared, a significant difference was found between the 2 groups, particularly in the early period of injury (at the 12th hour and on the 1st day), and this difference became highly significant on the 1st day. The course of IMA showed a similar pattern in the control and genistein groups, but a different pattern was observed in the trauma group. Although IMA plateaued in the trauma group on the 5th day, it plateaued on the 3rd day in the control and genistein groups (**Table 1**). IMA levels stabilized earlier in the genistein group than they did in the trauma group. In addition, at the 12th hour after the trauma, IMA levels in the control group showed a pattern comparable to those in the genistein group, but unlike that in the trauma group.

The thiol component containing sulfhydryl (-SH) is important for the antioxidative impact of oxidative processes. Thiol groups of some sulfur-containing amino acids are oxidized by reactive oxygen radicals and are converted reversibly into disulfide bonds. Levels indicated by NT are thiols that are normally found in plasma. The sum of the NTs and thiols formed by the reduction of disulfides is TT. Therefore, the increase in disulfide values represents an increase in the oxidant capacity, and the increase in thiol values represents an increase in the antioxidant capacity [25, 36,37,38]. Dynamic thiol/disulfide balance has been used frequently in clinical studies because of its antioxidant effect and its effect on apoptosis [5, 36,37,38]. In the present study, compared with the control group, there was a significant difference between the trauma and genistein groups, particularly on the 7th day (**Table 1**). There was no significant change between the disulfide values at the baseline and on the 7th day in the control group, whereas disulfide values increased in the trauma group, and showed a marked decrease in the genistein group compared with the baseline (**Table 1, Figure 3**). Oxidative capacity decreased in the genistein group. NT and TT levels fluctuated with temporal increases and decreases in the trauma and genistein groups. This may be because thiols require more time to achieve a steady state. However, the NT/TT ratio decreased in the control and genistein groups and slightly increased in the trauma group at the 12th hour. When the ratios at the 12th hour and those on the 7th day were compared, no change was observed in the control group, but the ratio decreased in the trauma group and increased in the genistein group (**Table 1**). Accordingly, although the TT and NT levels alone were not significantly different between the groups, based on the NT/TT ratio we concluded that there was increased antioxidant capacity in the genistein group.

Cox-2 is a conditionally-induced enzyme that is expressed in the event of inflammatory damage [39]. Cox-2 plays a role in the inflammation in secondary damage related to SCI. Cox-2 mRNA and protein expression were increased following SCI, and selective inhibition of Cox-2 led to improved functional outcomes, and neuronal cell death and neuro-inflammation were suppressed in experimental models [40]. In addition, Cox-2 expression decreases with the increase in antioxidant defense and decreases under oxidative stress [41, 42]. Genistein inhibits Cox-2 in laboratory studies [43]. The present study found a significant difference in Cox-2 expression between the genistein group and the control and trauma groups. A lower number of Cox-2 IR cells were found in spinal cords from rats in the genistein group than in those from the trauma and control groups (**Table 2, Figure 5**). This suggests that inflammation in the genistein group is suppressed compared with that in the trauma group. These results also suggest that genistein had an anti-inflammatory effect on the spinal cord. No overt histopathological difference was found between the groups. This highlights the importance of our immunohistochemical findings, because assessing the inflammatory response from HE staining alone is difficult in neural tissue.

Functional assessment of the rats showed that the baseline functional outcomes were worse in the trauma and genistein groups compared with those in the control group, but rats in the genistein group achieved better functional outcomes than those in the trauma group over time. Moreover, on the 5th day and thereafter, the significant difference between the control and trauma groups persisted, whereas a significant difference between genistein and the control groups was not found. This result showed that better functional outcomes were achieved in the genistein group.

The 7-day follow-up period in the present study was short, limiting its prediction of long-term outcomes of trauma. In addition, the induced model of spinal cord trauma is not a model of severe spinal cord trauma, limiting its prediction of severe SCI outcomes. The study is limited by using the BBB score alone to evaluate function. Although oxidative stress markers are considered to be appropriate for assessment in this model in rats, a problem is that they are highly variable in patients with multiple traumas, and may not be useful in clinical practice.

## Conclusions

Genistein has antioxidant, protective, and anti-inflammatory effects in an experimental model of SCI in rats. Further study of genistein is warranted as it shows potential as a therapeutic agent for the clinical treatment of SCI.
